# Chitosan nerve tube for primary repair of traumatic sensory nerve lesions of the hand without a gap: study protocol for a randomized controlled trial

**DOI:** 10.1186/s13063-015-1148-5

**Published:** 2016-01-26

**Authors:** Florian Neubrech, Sina Heider, Leila Harhaus, Berthold Bickert, Ulrich Kneser, Thomas Kremer

**Affiliations:** Department of Hand, Plastic and Reconstructive Surgery, Burn Center, BG Trauma Center Ludwigshafen, Plastic- and Hand Surgery, University of Heidelberg, Germany, Ludwig-Guttmann-Str. 13, D-67071 Ludwigshafen, Germany

**Keywords:** Nerve lesions of the hand, peripheral nerve surgery, nerve tube, sensory outcome

## Abstract

**Background:**

Complex peripheral nerve injuries of the hand include at least 300,000 cases per year in Europe. The standard treatment involves a microsurgical end-to-end suture of traumatic sensory nerve lesions of the hand without a gap. The objective of this study protocol is to evaluate whether the additional use of a chitosan nerve tube in primary repair of traumatic sensory nerve lesions of the hand without a gap has an effect on the recovery of sensitivity.

**Methods/Design:**

We planned a randomized double-blind controlled multicenter trial with a parallel group design in order to show superiority for the additional use of a chitosan nerve tube. This study will enroll 100 participants with traumatic sensory nerve lesions of the hand without a gap from three Trauma Care Centers. Participants will be randomized in a 1:1 ratio to primary microsurgical repair of the injured nerve with the additional use of a chitosan nerve tube or direct tension free microsurgical repair of the injured nerve alone. The static two-point discrimination of the injured finger after 6 months will be the primary outcome parameter.

**Discussion:**

In the proposed study, the additional use of a chitosan nerve tube for a primary microsurgical repair of traumatic sensory nerve lesions of the hand without a gap will be evaluated in a prospective randomized double-blind controlled multicenter trial for the first time to create the highest possible evidence for the procedure.

**Trial registration:**

ClinicalTrials.gov Identifier: NCT02372669.

Protocol Registration Receipt on 27 February 2015.

## Background

Complex peripheral nerve injuries of the hand include at least 300,000 cases per year in Europe [1]. In recent decades, considerable efforts have been made to support peripheral nerve regeneration but often with limited and minor success. At least one-third of patients will never regain normal sensitivity of the injured finger [2]. Subsequently, nerve dysfunction may lead to impaired function of the whole hand. This is especially true if the thumb as well as the index and small fingers are affected. Painful neuroma formation is frequently observed after peripheral nerve injuries of the hand. Sixty percent of these patients are observed with persistent pain lasting for years in up to 10 % [3–5].

Injured nerves do not spontaneously restore their function. Continuity of the nerve has to be re-established by microsurgical intervention. Standard treatment for traumatic sensory nerve lesions of the hand without a gap is an end-to-end suture, whereas nerve gaps are generally treated using autologous nerve grafts [6]. However, functional results often are disappointing, and painful neuroma formation is frequently observed after nerve repair. Moreover, autologous nerve grafting comes along with significant donor site morbidity [7]. To overcome this problem, alternative treatment options such as biosynthetic nerve grafts (little bio-absorbable implantable tubes) have been developed, primarily for the repair of short gap injuries. Nerve tubes should bridge the gap in this situation, protect the nerve from scar formation, and guide the regenerating axons to the distal stump [7]. The basic design of these nerve tubes is similar, but they are made of different absorbable biomaterials. For chitosan, a bioactive effect is reported [8–11].

This study should focus on the use of nerve tubes in addition to the primary microsurgical repair of traumatic sensory nerve lesions of the hand without a gap. So far, evidence for this approach is lacking. However, consolidated findings regarding tension in nerve repair and reconstruction of nerve gaps using chitosan tubes, as well as the biomaterial chitosan itself, are available to support the procedure.

Above all, tension in the repaired nerve prohibits the healing process [12]. The use of a nerve tube allows a tension-free suture. Possibly, that is the reason why nerve repair using a nerve tube bridging a gap of several millimeters was shown to have even better results than primary repair in injuries without nerve loss [13].

Promising clinical results have already been observed for nerve gap reconstruction using nerve tubes. The meta-analysis of Meek et al. summarizes the available literature and reports good and excellent results after 11 months in 75 % of the cases [14]. However, the authors still conclude that prospective randomized controlled trials with a systematic comparison of standard treatment and biosynthetic implants are lacking and that a systematic evaluation of only one biomaterial is required. Whether the repair of nerve lesions without a gap using a chitosan nerve tube can obtain comparable results is uncertain.

Chitosan, a derivative of chitin, is a biocompatible and biodegradable material and is similar to natural glycosaminoglycans. In vivo studies showed positive effects on the survival and orientation of the Schwann cells [8], as well as on the survival and differentiation of the neuronal cells [9, 10]. Furthermore, the substance itself seems to prevent painful neuroma [11]. Therefore, it potentially is the ideal material for nerve tube reconstruction. In this study, chitosan nerve tubes will be tested for use in treating traumatic sensory nerve lesions of the hand without a gap for the first time.

The assessment of sensory recovery after peripheral nerve surgery also remains challenging. Sensitivity of the hand is a complex process and can only be assessed with several tests addressing a variety of partial functions. Strictly speaking, the two-point discrimination (2-PD) is just an assessment tool for tactile gnosis. Tactile gnosis is the ability to recognize shapes and is a marker of functional recovery [15]. An age-related decline was shown in healthy individuals [16]. Based on that fact, nerve regeneration has often been postulated also to decrease with age [17], but this has not yet been proven. More importantly, interfering variables such as the distance from the lesion to the finger pulp exist. This distance was the only interfering variable that was shown in Meeks meta-analysis [14]. However, measuring the two-point discrimination provides a ratio of scaled values, making it particularly suitable for a scientific approach. The Semmes-Weinstein monofilament test, in contrast, assesses the ability for the perception of cutaneous pressure and is a marker for re-innervation in a stricter sense but does result in categories and thereby in a minor level of measurement [18].

The objective of this study protocol is to evaluate whether the additional use of a chitosan nerve tube in the primary microsurgical repair of traumatic sensory nerve lesions of the hand without a gap has an effect on convalescence and functional results.

### Hypotheses

#### Alternative hypothesis (H1)

The additional use of a chitosan nerve tube in the primary microsurgical repair of traumatic sensory nerve lesions of the hand without a gap will be superior compared with microsurgical repair alone.

#### Null hypothesis (H0)

The additional use of a Chitosan nerve tube in the primary microsurgical repair of traumatic sensory nerve lesions of the hand without a gap will be equal to or inferior to microsurgical repair alone.

## Methods/Design

### Design of the study and setting

To answer the question of whether the additional use of a chitosan nerve tube in primary microsurgical repair of traumatic sensory nerve lesions of the hand without a gap will improve the recovery of sensitivity, a randomized controlled multicenter trial with parallel group design was planned to show superiority for the additional use of a nerve tube. This study will enroll participants with traumatic sensory nerve lesions from three Centers: the BG Trauma Center Ludwigshafen (Ludwigshafen, Germany), BG Trauma Center Frankfurt am Main (Frankfurt am Main, Germany), and BG Bergmannsheil Bochum (Bochum, Germany). Trauma Center Ludwigshafen will be the executive study center.

### Screening

After being informed about the study and its potential risks, all individuals with traumatic sensory nerve lesions of the hand without a gap will be consecutively screened for eligibility until the recruitment period is over. Informed consent comprises a description of the procedures and objectives of the study and the follow-up period. Patients are informed that participation is completely voluntary and does not come with direct benefits and that denial does not come with disadvantages for the participant. The use by the trial of an already accredited and CE-certified product will be explained but in a blinded study setting. Potential adverse events regarding surgery in general and regarding the implant in particular are explained. The participants have to give their assent for blinded therapy and for the follow-ups. Participants are instructed to report any exceptional events immediately to the study center. Screening will be performed by younger physicians in the emergency ward who are not involved in randomization, surgery, or follow-up. A case report form will be prepared that will be used for the whole investigation.

### Recruitment

A two-phase recruitment will be conducted with a preoperative and intraoperative survey. After an individual has been found to be eligible by checking the preoperative inclusion and exclusion criteria, immediate operative treatment will follow. In the second phase of recruitment, the surgeon will explore the wounds and check the intraoperative inclusion and exclusion criteria. The surgeons are experienced physicians and are not involved in the screening or follow-up.

### Randomization

Finally, the enrolled participants will be randomized in a 1:1 ratio by alternating lists in the operating room to primary microsurgical repair with the additional use of a chitosan nerve tube or direct tension-free microsurgical repair of the nerve alone. Final enrollment and randomization following a fixed scheme will be performed by experienced surgeons who are not involved in the screening or follow-up. The CRFs will be accomplished for follow-up.

### Blinding

The kind of intervention is blinded for the participant and for the follow-up investigator, who was not involved in the surgery. In all study centers, medical postgraduates were employed for the follow-up examination. These raters are explicitly excluded from the screening, randomization, and operative procedure. The randomization list is kept locked in the operating room and is only available to the trial leaders and the surgeons for the process of randomization. After enrollment, only a running number on the CRFs will identify the participant’s position in the list. The rater only has access to the CRFs and not to the randomization list. Adherence to chronological randomization by the list will be monitored by an executive study center. The operation record will only include information about participation in the trial but will not include information about dedicated group. Unblinding is only permitted after dropout or after completion of the trial and can only be performed by the trial leaders.

**Fig. 1:**
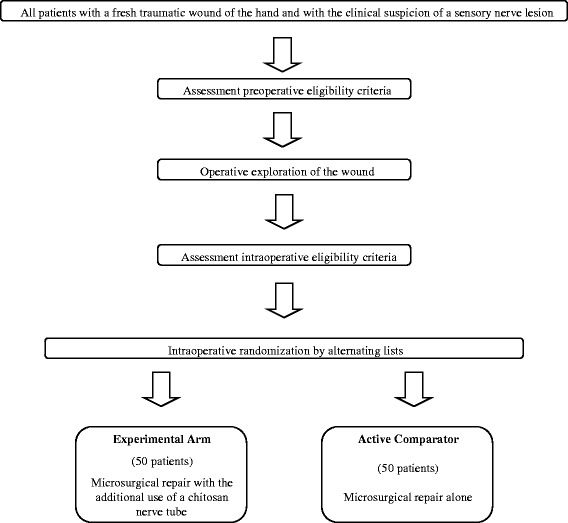
Study flowchart

Figure 1 shows the study flowchart.

### Participants

All individuals with a fresh traumatic wound of the hand and with the clinical suspicion of traumatic sensory nerve lesions of the hand without a gap will be consecutively screened for eligibility during the preoperative and intraoperative periods.

Preoperative inclusion criteria are as follows:

1. Clinical suspicion of traumatic sensory nerve lesions of the hand without a gap (lesion from the distal area of the carpal tunnel to the end finger joint with complete loss of a nerve-specific receptive field of the finger).
2. Age between 18 and 67 years.
3. Trauma occurred in the previous 72 h.
4. Signed informed consent.

Preoperative exclusion criteria are as follows:

1. Amputated or avascular fingers.
2. Infection of the wound.
3. Known pre-existing impaired sensibility of the injured finger or the opposite side.
4. Allergy to chitosan.
5. Pregnancy.
6. Known immunodeficiency.
7. Participation in other trials.

Intraoperative inclusion criteria are as follows:

1. Verification of a traumatic sensory nerve lesion of the hand without a gap.
2. Nerve can be sutured in end-to-end fashion.

Intraoperative exclusion criteria are as follows:

1. Avascular fingers.
2. Multiple nerve lesions, which cannot be randomized uniformly.
3. Nerve injuries with a gap.

### Outcomes

The primary study objective is the recovery of sensitivity. The assessment will follow the guidelines developed by Rosén [19]. Recovery of sensibility will be assessed by static two-point discrimination (tactile gnosis) and the Semmes Weinstein method (sensory re-innervation). The static two-point discrimination (2PD) after 6 months will be the primary outcome parameter. The 2PD does characterize the sensory capability of the injured finger and is a standard method used in the past for clinical assessment of a variety of artificial nerve tubes [14]. The distance between the lesion and the finger pulp will be attributed in the statistical analysis.

Sensory re-innervation, DASH-score, grip strength, total active range of motion, pain, cold intolerance and hypersensitivity, and the appearance of neuroma will be secondary outcome parameters.

All outcome parameters are frequently used in hand surgery research.

### Interventions

Surgery will be performed in plexus anesthesia or under general anesthesia, depending on the individual’s request and anesthesiologist’s decision, with the use of a tourniquet in a bloodless field. The wound first will be cleaned by flushing and surgical debridement and then explored via Brunner’s incisions [20] using loupes. The injured nerve will be inspected and checked for intraoperative eligibility. Final enrollment and randomization is carried out intraoperatively. Further treatment depends on the dedicated group. If more than one nerve is injured, all the nerves of one individual will be treated in the same fashion. The kind of intervention is blinded for the participant.

#### Experimental group

Participants who are allocated to the experimental group will be treated with an additional chitosan nerve tube. The injured nerve will be exposed and neurolysed for a distance of 2 cm on each side of the lesion. From this point forward, a microscope will be used. The injured ends of the nerve will be sparingly freshened. The chitosan implant will be pre-soaked for 10 minutes in saline. Afterward, the implant (with a length of 1 cm and an inner diameter of 2.1 mm) will be imposed on the proximal nerve stump. A complete tension-free epineural coaptation of the nerve ends will follow, using several 9–0 USP (United States Pharmacopoeia-System) nylon micro-sutures (German Sutures SAS, La Barbiniere, Beaurepaire, France).

Finally, the nerve tube will be positioned at the site of the suture and fixed to the epineurium with a single stitch on each side using a 9–0 USP (United States Pharmacopoeia-System) nylon micro-suture. The use of the chitosan-based nerve tube is already an authorized and CE-certified German medical product (Reaxon® Nerve Guide, Medovent, Mainz, Rheinland-Pfalz,  Germany)

#### Active comparator group

Participants who were allocated to an active comparator group will be treated by standard treatment without using an additional chitosan nerve tube. The injured nerve will be exposed and neurolysed for a distance of approximately 2 cm on each side of the lesion. A microscope will be used from this point onward. The injured ends of the nerve will be sparingly freshened. A tension-free epineural coaptation of the nerve ends using several 9–0 USP (United States Pharmacopoeia-System) nylon micro-sutures will complete the nerve repair (German Sutures SAS, La Barbiniere, Beaurepaire, France).

Other concomitant injuries will be treated independently of the study protocol in the same session.

### Follow-up

During follow-up, the prepared CRFs will be used by the rater. These CRFs will already include age, gender, profession and current activity, mechanism of the accident, handedness, medical history concerning the hands, date and time of injury, injured side, injured finger, injured nerve, concomitant trauma, and distance from lesion to finger pulp.

Follow-up examinations will be performed after 3, 6, 12, and 24 months. The parameters described below will be measured at 3, 6, 12 and 24 months. Each assessment will take approximately 45 min. In all study centers, medical postgraduates have been employed for the follow-up examination. These raters are explicitly excluded from the screening, randomization, and operative procedure.

#### Tactile gnosis: static two-point discrimination (2PD)

The ability of perception of either one or two points of touch will be assessed using a compass (NCD Medical/Prestige, Los Angeles, CA, USA). Participants will sit in front of the rater with the dorsum of the hand on the table. Test will be performed on the halves of finger pulps with preoperatively failed nerve-specific receptive fields and on the correlating finger pulps of the contralateral hand. Pressure is defined by self-weight of the compass (10 g). Two points will be applied in a longitudinal direction with decreasing distance between them until they are perceived as one point. Final distance (in mm) will be the final value. The 2PD measured at 6 months will be the primary outcome parameter; the other values measured at 3, 12 or 24 months will be regarded as secondary outcome parameters.

#### Sensory re-innervation: Semmes Weinstein method

A monofilament is pressed against the halves of the finger pulps with preoperatively failed nerve-specific receptive fields and on the correlating finger pulps of the contralateral hand starting with the thinnest filament. Depending on the response, thicker ligaments are applied until the participant feels the pressure. Six kit monofilaments (Patterson Medical, Warrenville, IL, USA) will be used. Value is measured in grades as follows:

0: not testable
1: filament 6.65 = perception of deep pressure
2: filament 4.56 = no protective sensation
3: filament 4.31 = diminished protective sensation
4: filament 3.61 = diminished perception of light touch
5: filament 2.83 = normal perception of light touch

The sensory re-innervation measured by the Semmes Weinstein method is a secondary outcome parameter.

#### Disabilities of the arm, shoulder, and hand (DASH)

Individuals will self-report their disabilities in activities of daily living by using a DASH (disabilities of the arm, shoulder, and hand) questionnaire [21] in its validated German version [22]. The questionnaire consists of 30 items regarding the requirements of daily life. Optional sport and music modules also exist that are not part of the scoring system. Individuals will assess each item using a scoring system with a range between 1 (no problem with the activity) and 5 (activity is not possible any more). Which hand is used for the activity is not relevant. The assigned values for all completed responses are simply summed and averaged, producing a score out of five. This value is then transformed to a score out of 100 by subtracting one and multiplying by 25, resulting in a DASH score range of 0 to 100. A higher score indicates greater disability. The disabilities of the arm, shoulder, and hand questionnaire (DASH) is a suitable indicator for the burden of the disease by handicaps of the hand [21]. *The DASH*-*score* will be a secondary endpoint.

#### Grip strength

Grip strength will be measured with a Jamar dynamometer (Sammons Preston Inc., Bolinbrook, IL, USA) in stage II. Participants will sit in front of the rater with elbows close fitting to the body and wrists in neutral position. Participants will be requested to press the dynamometer as hard as they can. Three trials with each hand will be carried out. The highest trial will be valued and will be compared with the uninjured hand. Grip strength measured as percentage compared to the contralateral hand will be a secondary endpoint.

#### Total active range of motion

Total active range of motion of the injured fingers and of the corresponding contralateral fingers will be measured with a goniometer for small joints (Fabrication Enterprises, White Plains, NY, USA). Participants will sit in front of the rater, elbow on a table, palm of the hand upturned. The arms of the goniometer will be laid along extension side. The total active range of motion is calculated by summation of the range of motions for flexion and extension of all joints of the concerned finger. The total active range of motion will be a secondary endpoint.

#### Pain, cold intolerance, and hypersensitivity

Individuals will be asked to self-report pain, cold intolerance and hypersensitivity experienced in the last week on a 100-mm visual analog scale, ranging from 0 (no symptoms) up to 10 (maximum of symptoms) by drawing a point. Visual analog scales are well validated for the measurement of acute and chronic pain [23]. Pain, cold intolerance, and hypersensitivity will be secondary endpoints.

#### Appearance of neuroma

The existence of a neuroma will be assessed by local percussion of the injured nerve site. Electrifying pain will be valued as suspicious for a neuroma. These findings will be verified by neurosonography (linear device, 14 MHz frequency). A final data report will include the count of clinical suspicious neuroma and the count of verified neuroma by sonography. The appearance of neuroma will be a secondary endpoint.

Table 1 summarizes study outcome measures.

**Table 1:** Study outcome measures

Item	Outcome measurement [unit]	Time point of measurement	Primary/secondary outcome parameter?
Tactile gnosis**	Static 2-point-discrimination [mm]	at 6 months postoperatively**	primary**
Tactile gnosis**	Static 2-point-discrimination [mm]	at 3, 12, and 24 months postoperatively**	secondary**
Sensory re-innervation	Semmes Weinstein method [grading]	at 3, 6, 12, and 24 months postoperatively	secondary
Disabilities in activities of daily living	DASH questionnaire [score]	at 3, 6, 12, and 24 months postoperatively	secondary
Grip strength	Jamar dynamometer [kg; percentage of contralateral side]	at 3, 6, 12, and 24 months postoperatively	secondary
Motion	Total active range of motion [degree]	at 3, 6, 12, and 24 months postoperatively	secondary
Pain, cold intolerance, hypersensitivity	Visual analog scales [points out of ten]	at 3, 6, 12, and 24 months postoperatively	secondary
Appearance of neuroma	Clinical examination and sonography [count]	at 3, 6, 12, and 24 months postoperatively	secondary

** Tactile gnosis measured by static two-point-discrimination 6 months postoperatively will be considered as the primary outcome parameter. Tactile gnosis** measured by the static two-point discrimination at 3, 12, and 24 months postoperatively will be considered as the secondary outcome parameters

In addition, adverse events (any type of revision surgery, incidence of postoperative hematoma, deep wound infections, disturbances of scar formation such as hypertrophic or instable scars) will be recorded during the hospital stay (approximately 5 days) and retrospectively in the follow-ups. Revision surgery of one affected site will be considered as an adverse event even in cases of multiple nerve injuries. Furthermore, the number of pre- and post-randomization dropouts will be registered and analyzed in a descriptive statistic.

### Sample size/power calculation

Assumptions for the primary outcome parameter of the standard treatment can be made from the literature [2, 14] and from our own patients. The mean of 2-PD after 6 months is approximately 8 mm with a standard deviation of 3 mm. A decrease of 2 mm in the 2-PD would be clinically relevant [16] and is assumed for the experimental intervention. Using a two-sided *t*-test with a level of 0.05 and a power of 80 %, 37 individuals per group will be required in order to show superiority. In order to compensate for loss to follow-up, 50 individuals per group will be randomized.

Before implementing the trial design, we analyzed the computerized medical records of our patients retrospectively, to determine how many individuals would have fit our scheme of inclusion and non-inclusion criteria per year. We discovered that we could have included at least 50 individuals per year in the last 3 years. With respect to the unequal number of hand surgical cases in the three centers, the intention is to enroll 100 individuals per year: 50 individuals in the BG Trauma Center Ludwigshafen, 25 individuals in the BG Trauma Center Frankfurt am Main, and 25 individuals in the BG Trauma Center Bochum. The study is intended to take place over a period of 36 months: 12 months for recruitment of all individuals, 18 months from the beginning of the trial for completion of the primary outcome parameter, and 36 months from the beginning of the trial to the completion of all follow-ups.

### Statistical analysis

The primary endpoint will be tested in the per-protocol set via an analysis of covariance with center as factor and distance between lesion and finger pulp as covariate. The test in the full analysis set is to be considered as the sensitivity analysis. Secondary endpoints will be described statistically without confirmatory analysis. Adverse events will be listed with detailed information concerning sort, timing, severity, and mandatory countermeasures.

### Data collection and monitoring 

Data will be collected in standardized CRFs according to European standards (DIN EN ISO 14155) and Good Clinical Practice recommendations. CRFs will be transmitted electronically to the executive study center in Ludwigshafen and will be checked there for integrity, quality, and consistency. The executive study center will also ensure standardization of the registry process, operative procedure, and follow-up in all participating centers by periodic monitoring. Furthermore, written instructions and a course of instruction will be provided to each investigator. Data will be collected and analyzed in the executive study center. CRFs will also used for reporting dropouts and for reporting adverse events.

### Ethical considerations

This study is being conducted in accordance with the principles of the Declaration of Helsinki and Good Clinical Practice guidelines. The study was approved by a German Ethics Committee (Ethics Committee of Rhineland-Palatinate, Mainz, Germany). Prior to randomization, written informed consent will be obtained from all participants. No expenses will be paid at all.

## Discussion

Standard therapy for traumatic sensory nerve lesions of the hand includes a direct tension-free microsurgical repair for lesions without a gap and autologous nerve grafting from another body region for lesions with a gap [6]. Alternatively, nerve gaps are increasingly reconstructed using nerve tubes to prevent donor site morbidity. A recent meta-analysis shows good and excellent clinical results for this approach [14]. However, prospective controlled single trials are still lacking, and hardly any systematic comparison of standard treatment and the use of nerve tubes are available [14]. The proposed study will compare standard treatment and the use of nerve tubes in a prospective controlled trial setting. Some hand surgery departments also use nerve tubes in addition to a direct nerve suture for lesions without a gap in hopes of improving the results, but such an effect has not yet been proven. Therefore, the major study objective of this trial will be to answer the question of whether the additional use of a chitosan nerve tube in primary microsurgical repair of traumatic sensory nerve lesions of the hand without a gap will improve the recovery of sensitivity. The results will have a direct implication on this common, but as of yet scientifically unproven, practice. A potential study limitation is an example of risk of selection bias because of the quasi-randomization method of using alternating lists. This limitation is due to the emergency surgery setting of the study. Of course, the determined power of 80 % power does also come along with a risk for type II errors of 20 %, but that is conventional for most medical trials. In addition, there is experimental evidence that the use of a chitosan nerve tube may improve functional outcome and reduce the incidence of a painful neuroma [7–11, 24, 25]. The proposed study will test the chitosan-based implant in a clinical setting. If we obtain promising results, we will expand the use of the chitosan nerve tube to use in further studies for traumatic sensory nerve lesions of the hand with a gap as an alternative to autologous nerve grafting and for traumatic nerve lesions of the forearm that include motor axons and therefore have an even more significant burden of disease.

### CONSORT statement

The authors hereby declare that final report will follow CONSORT as well as its extension to nonpharmacological interventions.

### Trial status

The study is currently enrolling participants.

## Abbreviations

CE: Mark of conformity of the European Union
CRF: case report form
DASH: Disabilities of the Arm, Shoulder and Hand
DIN: German Institute for Standardization
EN: European Norm
FESSH: Federation for European Societies for Surgery of the Hand
ISO: International Organization for Standardization
USP: United States Pharmacopoeia-System
2PD: two-point discrimination.
